# Advances in Infectious Bursal Disease Virus Vaccines—A Review

**DOI:** 10.3390/microorganisms13122801

**Published:** 2025-12-09

**Authors:** Weiwei Wang, Jiafeng Wu, Nansong Jiang, Qizhang Liang, Rongchang Liu, Qiuling Fu, Guanghua Fu, Tianchao Wei, Chunhe Wan, Longfei Cheng, Yu Huang, Xiumiao He, Ping Wei, Hongmei Chen

**Affiliations:** 1 Fujian Key Laboratory for Control and Prevention of Avian Diseases, Fujian Industry Technology Innovation Research Academy of Livestock and Poultry Diseases Prevention and Control, Institute of Animal Husbandry and Veterinary Medicine, Fujian Academy of Agricultural Sciences (FAAS), Fuzhou 350013, China; wangweiweihn@163.com (W.W.); 17660733329@163.com (J.W.); nansongjiang@126.com (N.J.); qzl1181@zuaa.zju.edu.cn (Q.L.); liurongc@foxmail.com (R.L.); qiulingfu0822@163.com (Q.F.); fuyuan163@163.com (G.F.); chunhewan@126.com (C.W.); lf396@139.com (L.C.); huangyu_815@163.com (Y.H.); 2 Institute for Poultry Science and Health, Guangxi University, Nanning 530004, China; tcwei88@126.com (T.W.); weiping@gxu.edu.cn (P.W.); 3 Guangxi Key Laboratory for Polysaccharide Materials and Modifications, School of Marine Sciences and Biotechnology, Guangxi University for Nationalities, Nanning 530008, China

**Keywords:** infectious bursal disease virus, attenuated vaccine, inactivated vaccine, immune complex vaccine, subunit vaccine, recombinant vector vaccine, reverse genetics vaccine, DNA vaccine, mRNA vaccine, self-amplifying vaccine

## Abstract

Infectious Bursal Disease (IBD) is an immunosuppressive viral disease caused by the Infectious Bursal Disease Virus (IBDV). It primarily affects young chickens, targeting the bursa of Fabricius, and poses significant economic threats to the poultry industry. To date, in addition to strict biosecurity measures, large-scale immunization is the optimal strategy and effective method to prevent and control IBDV infection. The emergence of new variant strains has made it more urgent to develop new vaccination strategies against IBD. Over the past few decades, many high-quality vaccines have been available on the market for the control of IBD, which can provide solid protection against the infections and diseases caused by classic IBDV to very virulent IBDV that had been continuously evolving and were endemic worldwide. However, viruses are not static. As they continue to circulate and evolve in the fields, novel antigenic variant viruses have been emerged in the last few years, and vaccines need to keep up with their pace. Collectively, this review summarizes the strategic evolution of IBDV vaccines from traditional methods to cutting-edge molecular platforms, providing promising strategies for developing the next-generation vaccines with higher safety, efficacy, and the ability to keep pace with the antigenic drift in IBDV.

## 1. Introduction

Infectious bursal disease (IBD), is an acute and highly contagious immunosuppressive disease of young chickens caused by infectious bursal disease virus (IBDV) [1]. IBDV belongs to the family *Birnaviridae* and the genus *Avibirnavirus*, with a bi-segmented double-stranded RNA genome designated segments A and B. Segment A (~3.3 kb) encodes the nonstructural protein VP5 (~17 kDa), the capsid protein VP2 (~40 kDa), the scaffold protein VP3 (~32 kDa), and the viral protease VP4 (~28 kDa). VP2 is the major structural and host-protective immunogen, which are implicated in cell tropism, virulence, and antigenic variation [2]. Segment B (~2.8 kb) contains a single open reading frame encoding the RNA-dependent RNA polymerase VP1 (~90 kDa), which plays a crucial role in viral replication, genetic evolution, and virulence [2]. The virus primarily targets and destroys immature B lymphocytes in the bursa of Fabricius (BF), leading to severe immunosuppression, secondary infections, and reduced efficacy of vaccination against other pathogens [2]. Since its discovery in the 1960s in the United States, IBD has become one of the most important diseases in the poultry industry, with high morbidity, considerable mortality, and substantial economic losses worldwide. Two serotypes of IBDV have been identified: serotype 1, which is pathogenic, and serotype 2, which is nonpathogenic [3,4]. Over time, serotype 1 virus has evolved into multiple pathogenic strains, including classical (cIBDV) [5], very virulent (vvIBDV) [6], and antigenic variant (avIBDV) strains [7,8,9]. Similarly to other segmented RNA viruses, co-circulation of different types of IBDV strains in the field, combined with long-term immune pressure, facilitates viral evolution and the emergence of antigenic variants capable of escaping host immunity [10].

**Figure 1:**
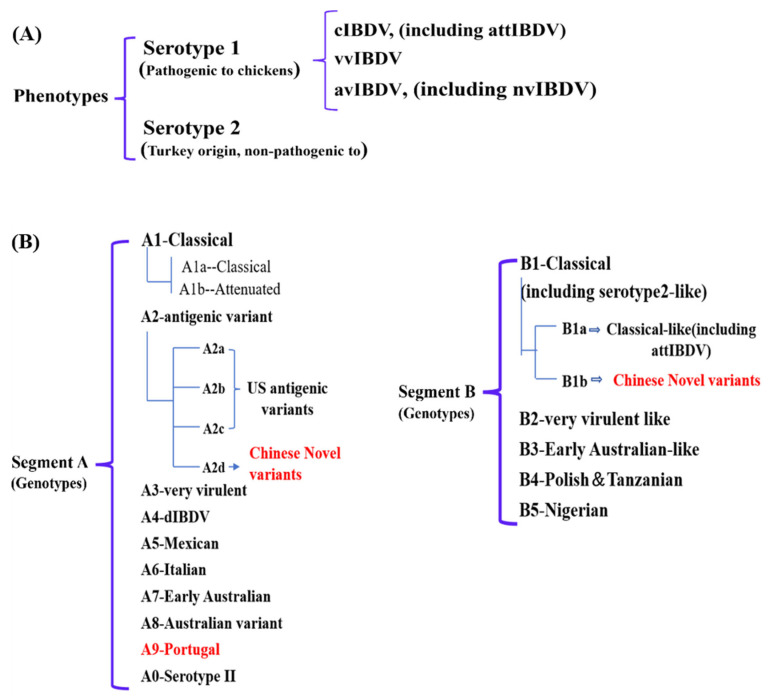
The latest phenotypes (**A**) and genotypes (**B**) classification of IBDV. The red font indicates the newly emerged strains in recent years.

Owing to the rapid genetic variation occurring within the hypervariable region of the VP2 gene (vVP2) in field isolates, Michel and Jackwood [11] proposed a classification framework that divides IBDV into seven primary genogroups: classical, antigenic variant, vvIBDV, dIBDV, variant/classical recombinant, Italian (ITA) and Australian strains. This genotyping strategy generally corresponds to the earlier phenotype-based categorization. Nevertheless, accumulating evidence indicates that the pathogenic characteristics and evolutionary dynamics of IBDV are determined not only by mutations in vVP2, but also by genetic changes in the VP1 gene on segment B of the viral genome. A more comprehensive dual-segment genotyping system was subsequently introduced by Islam et al. [12], which categorized IBDV into nine genogroups of segment A (A1, classical; A2, US antigenic variant; A3, very virulent; A4, dIBDV; A5, atypical Mexican; A6, atypical Italian; A7, early Australian; A8, Australian variant and A0, serotype 2) and five genogroups of segment B (B1, classical-like; B2, very virulent-like; B3, early Australian-like; B4, Polish & Tanzanian and B5, Nigerian). Later, Y-L. Wang et al. [13] proposed a revised scheme that largely aligns with the classification of Islam et al. Our group found that the VP1 gene of the nvIBDV was different from the early classical-like (B1), so it was proposed to divide it into B1a (classical like) and B1b (Chinese novel variants), that is, the nvIBDV was a novel genotype (A2dB1b) (Figure 1) [10].

When selecting IBDV vaccines, poultry veterinarians must address key factors including maternal immunity, litter management, and desired protection endpoints [14,15,16]. Maternally derived antibodies (MDA) are crucial for the early post-hatch period protection but can interfere with vaccine efficacy [16,17]. Veterinarians must evaluate MDA levels, which are influenced by the vaccination protocols of breeder hens. In regions with well-established breeder vaccination programs, MDA provides early protection, but excessive MDA can impede active immunity in chicks, necessitating the use of vaccines compatible with MDA interference, such as immune complex (Icx) or recombinant vector vaccines [18,19]. Litter management also impacts vaccine choice, particularly in systems with reused litter where IBDV can persist between flocks, posing an early exposure risk. In these cases, vaccines offering early protection, like Icx or recombinant vectored vaccines, are essential. In contrast, systems with single-flock litter replacement and low environmental challenge may rely on breeder vaccination and biosecurity measures, reducing the need for active vaccination in broilers. Finally, vaccine selection must align with specific protection goals, including the timing, duration, and type of immunity required [20]. Live attenuated vaccines provide rapid immunity but may be less effective in high-MDA conditions, while inactivated vaccines, though slower to induce immunity, offer longer-lasting protection [14,15,16]. Vaccine choice should also account for circulating field strains and emerging variants [13].

**Figure 2:**
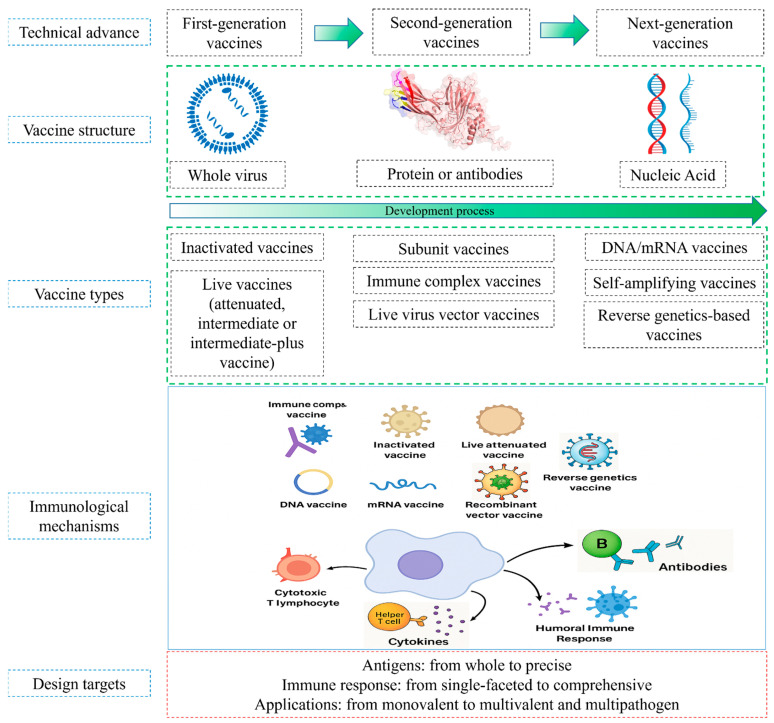
IBDV vaccine platforms, current advances and immunological mechanisms.

Vaccination remains the cornerstone of IBD control, with a variety of vaccines in use, including attenuated, inactivated, Icx, and recombinant viral vector vaccines, currently in use (Table 1). However, although these approaches have provided substantial protection, their efficacy is challenged by MDA interference, antigenic drift, and the potential for immunosuppression or reversion to virulence [14,15,16]. The continuous evolution of IBDV has stimulated the development of next-generation vaccines, including recombinant VP2-based subunit formulations, viral vector platforms, and nucleic acid vaccines, designed to improve safety, broaden cross-protection, and enhance immunogenicity. This review aims to summarize these research advances, focusing on the development of next-generation vaccines that promise higher safety, efficacy, and the ability to keep pace with the antigenic drift of IBDV (Figure 2).

## 2. Conventional Live IBDV Vaccines

Live vaccines are prepared from attenuated or avirulent pathogens obtained through induced mutagenesis or natural selection. These vaccines can mimic natural infection by replicating within the host and inducing strong immune responses, including both cellular and humoral immunity. At present, live vaccines can be classified into three types, including mild, intermediate or intermediate-plus, and virulent [17,18]. Mild vaccines often show poor efficacy in chicks with MDA, since MDA interferes with viral replication. Therefore, waiting for an appropriate period of time until MDA levels weaken is crucial to achieve reliable immunity [19,20]. Intermediate and intermediate-plus vaccines (also known as “hot” vaccine) provide superior protection against field strain infections and can overcome relatively high levels of MDA; however, they may also induce BF lesions and subsequent immunosuppression [21,22]. Additionally, recent studies indicate that such vaccines do not fully protect against infection with vvIBDV or novel variant IBDV strains (nvIBDV) [23,24]. Virulent vaccines are rarely used in the industry due to their potential to cause severe BF damage in vaccinated flocks. Although live vaccines do not require adjuvants and can effectively stimulate immune responses, making them suitable for large-scale immunization in poultry, their safety and efficacy remain concerns. For example, genetic reassortment or homologous recombination between vaccine and field strains has been reported [10,25,26], and prolonged circulation of live vaccines in flocks can, in rare cases, result in selection toward increased virulence or altered antigenicity; these phenomena underline the need for careful vaccine strain selection and surveillance. Generally, live IBDV vaccines used in the poultry industry are derived from classical strains that are serially passaged in cells or chicken embryos to attenuate virulence while retaining strong immunogenicity [2]. Okura et al. developed an attenuated live vaccine (IBD-CA) from the cIBDV Lukert strain via chicken embryo fibroblast (CEF) culture, which provided better protection against vvIBDV challenge than the rHVT-VP2 vaccine [27]. Similarly, Leng et al. demonstrated that the attenuated live strain W2512 induced high levels of IBDV antibodies and protected chickens against nvIBDV infection via a placeholder effect; however, it also caused severe BF atrophy in both SPF chickens and commercial yellow-feathered broilers [28].

## 3. Conventional Inactivated IBDV Vaccines

The inactivated IBD vaccines are typically formulated as water-in-oil emulsions and often combined with multiple antigens, such as inactivated whole virus, recombinant virus, or viral subunits, etc. These vaccines are widely used in the industry due to their high safety and non-infectious [14]. Compared to live IBDV vaccines, inactivated vaccines can induce high levels of antibodies but generally elicit limited cellular immune responses unless combined with highly efficient adjuvants and repeatedly immunized. Rautenschlein et al. found that IBD inactivated vaccines can also stimulate IBDV-specific T-cell responses [29]. Generally, to achieve effective protection, inactivated IBDV vaccines need to contain high concentrations of optimized antigens, which can stimulate strong antibody responses in chickens and protect offspring against IBDV strains [30]. Wang et al. evaluated the protective efficacy of oil-emulsified inactivated vaccines (OEVs) prepared from two nvIBDV isolates, QZ191002 strain (A-nv/B-nv) and YL160304 (A-nv/B-HLJ0504-like), against nvIBDV challenge. Their findings demonstrated that a single priming dose of the commercial vaccines (without any antigen of nvIBDV) provided incomplete protection (40–60%), which was only partially improved to 60–80% with a booster. Crucially, only the homologous OEVs conferred complete protection (100%) [31]. Cui et al. demonstrated that recombinant chicken interleukin-7 (IL-7), as a potent adjuvant, can enhance the immunogenicity and protective efficacy of inactivated IBDV vaccines [32]. Many breeding companies use high-quality inactivated vaccines to immunize breeder hens before laying to provide passive immunity to chicks via MDA. Several high-quality inactivated IBDV vaccines have been developed, and the “prime-boost” immunization strategy is considered the most effective approach for inactivated vaccine immunization in field applications.

## 4. Immune Complex Vaccines

Immune complex vaccines (Icx) against IBDV were first developed in the late 1990s. These vaccines are composed of an intermediate-plus IBDV strain mixed with IBDV-specific antibodies obtained from the hyperimmunized serum, forming a virus–antibody complex (IBD-Icx) [33]. Notably, IBD-Icx vaccines have demonstrated efficacy even in the presence of high levels of MDA [34], whether administered via in ovo vaccination at day 18 using commercial automated egg-injection systems or via subcutaneous injection in one-day-old chicks, providing protection against both vvIBDV and avIBDV [35]. During the challenge, the experimental efficacy of the Icx vaccines was identical to or better than that induced by vaccination with live IBDV vaccines. Ivan et al. reported that viruses were first detected in the bursa of SPF chickens vaccinated with IBD-Icx at day 14 post-vaccination and were subsequently observed in chickens with MDA against IBDV between days 17 and 21 post-vaccination [36]. Due to IBD-Icx will initially remain bound to the surface of follicular dendritic cells present in the bursa of Fabricius and spleen, until maternal-derived antibodies (MDA) decrease to a point where the moderately virulent live vaccine can begin to replicate in the bursa without causing bursal atrophy or immunosuppression.

A major breakthrough has been the shift from polyclonal antisera to the use of recombinant neutralizing antibodies. This shift addresses critical issues of batch-to-batch variability and scalability associated with traditional hyperimmune serum. By cloning and expressing monoclonal antibodies (mAbs) with defined neutralizing epitopes, researchers can now create standardized, well-characterized Icx vaccines. This precision allows for the fine-tuning of the antigen-to-antibody ratio, optimizing the complex’s size and stability to ensure efficient targeting and retention on follicular dendritic cells in lymphoid tissues. More recently, recombinant neutralizing antibodies have been developed for use in IBD-Icx vaccines [37]. Another promising frontier is the integration of Icx technology with nanoparticle delivery systems. The ultimate goal is to create “smart” vaccines that not only safely bypass pre-existing immunity but also actively direct the immune system towards a more potent and durable response.

## 5. Live Virus Vector Vaccines

Recombinant live virus vector vaccines are created by inserting the gene encoding the target antigen into harmless or attenuated bacteria or viruses, generating recombinant strains that replicate within the host. As these strains replicate, the target gene is expressed on a large scale, stimulating a protective immune response against both the vector and the target pathogen. The first-generation recombinant live virus vector vaccine for IBDV was developed in the early 1990s using fowlpox virus (FPV) expressing the IBDV VP2 protein (*r*FPV-VP2) [38]. This vaccine successfully induced IBDV-specific antibodies and protected chickens against IBDV challenge. However, its protection was less effective compared to traditional IBDV inactivated oil-emulsion vaccines [39]. A significant advancement was made by Eldaghayes, who developed the fpIBD1:IL-18 recombinant strain, which simultaneously expresses the IBDV VP2 protein and chicken interleukin-18 (IL-18). This recombinant vaccine provided complete protection against the IBDV F52/70 challenge [40]. Another recombinant vector vaccine was developed using Herpesvirus of turkey (HVT) as the vector [41]. Two commercial *r*HVT-VP2 vaccines, which express the VP2 protein from IBDV strains Faragher 52/70 and Variant E, were developed and licensed for international use in 2007 and 2015, respectively [42]. High levels of MDA have been confirmed not to interfere with the immune effect of the HVT-VP2 vaccine [43], and vaccination via in ovo or one day-old subcutaneous injection has proven to be safe and effective [44]. In addition, Hulten et al. developed a HVT-ND-IBD vaccine based on HVT vector, which enabling simultaneous protection against ND, IBD, and Marek’s disease virus (MDV) [45]. In addition to HVT, other viruses have been used to develop recombinant vector vaccines for IBDV [46], including MDV [47], NDV [48], avian adenovirus [49], semliki forest virus [50], baculoviruses [51] and T4 bacteriophage [52,52].

## 6. Subunit Vaccines

The capsid protein VP2 of IBDV has been the focus of subunit vaccine development due to its role as the primary host-protective antigen [30]. Multiple prokaryotic and eukaryotic expression systems, including *Escherichia coli* [53], yeast [54], baculovirus [55], *Lactococcus lactis* [56], and plant expression systems [57,58], have been developed for expressing and purifying the VP2 protein. Rong et al. developed a subunit vaccine using *E. coli* BL21/pET28a-VP2, which provided 90–100% protection in immunized chickens and has been widely applied for the IBD control [53,59]. Ji et al. expressed the IBDV VP2 protein in *E. coli* to develop an effective virus-like particle (VLP) vaccine and found that combining IBD VLPs with adjuvants significantly enhanced the vaccine’s immunogenicity [60]. Wang et al. successfully developed self-assembling sub-virus-like particle (sVLP) using *Pichia pastoris* and demonstrated that these vaccines induced high levels of IBDV-neutralizing antibodies, providing full protection against vvIBDV challenge [61].

Several other studies have focused on improving vaccine efficacy through different approaches. Pitcovski et al. achieved complete protection against IBDV using a yeast-expressed VP2 protein [62]. Martinez-Torrecuadrada et al. compared the immunogenicity and protective efficacy of various VP2 capsid structures and found that VP2 capsids elicited the strongest neutralizing response, followed by polyprotein-derived mixture and VPX nanotubes [51]. Liu et al. expressed a fusion protein of VP2 and chicken interleukin-2 (IL2) using an insect expression system, which significantly enhanced the immunogenicity of the VP2 protein [63]. Wang et al. used phage display technology to develop a multi-epitope protein vaccine (*r*5EPIS) with monoclonal antibody-binding peptides, which provided 100% protection in chickens challenged with vvIBDV GX8/99 [64]. More recently, Wang et al. developed a VP2 subunit vaccine using *Lactococcus lactis* to express VP2 from an nvIBDV, inducing unique neutralizing antibodies and providing 100% protection in immunized chickens [56]. Wu et al. developed an edible plant-derived IBDV-VP2 subunit vaccine, which stimulated chickens to produce specific antibodies following oral immunization with crude leaf extract [57]. Subsequently, Wu et al. developed an edible vaccine based on rice plants expressed VP2 protein specifically in seeds [65], which induced IBDV-specific neutralizing antibodies, with antibody titers increasing in a dose-dependent manner [66].

## 7. Reverse Genetics-Based Recombinant IBDV Live Vaccines

Advances in reverse genetics systems have greatly facilitated the generation of attenuated IBDV through targeted genomic modifications, providing potential candidates for vaccine development. Traditional attenuated vaccine strains are usually obtained by serial passage in cell culture or SPF embryos, or through natural isolation. However, these methods are often time-consuming, unpredictable, and particularly unsuitable for nvIBDV. Reverse genetics provides a more precise and efficient alternative. Based on these findings, several research groups have generated recombinant or chimeric IBDVs with attenuated phenotypes and strong immunogenicity. For example, Mosley et al. demonstrated that an IBDV rescued efficiently with 3′ authentic RNA sequence induces humoral immunity without BF atrophy but elicited stronger antibody responses as early as 7 days post-infection [67]. Similarly, mutation of the VP5 start codon yielded VP5-deficient mutants that retained replication competence, caused no clinical signs or lesions associated with the parental virus [68], yet induced comparable levels of IBDV-neutralizing antibodies [69]. Consistently, Qin et al. showed that VP5-deficient vvIBDV strains provided strong protection against wild-type vvIBDV challenge in chickens [70]. Boot et al. generated attenuated chimeric viruses by substituting the VP3 C-terminal region of serotype II with the corresponding region from serotype I [71]. Mundt et al. constructed a chimeric IBDV by substituting VP2 and VP4 of a classical vaccine strain with those from a variant strain, producing a virus with replication capacity equivalent to the classical vaccine strain but capable of inducing neutralizing antibodies against both classical and antigenic variant strains [72]. Likewise, Boot et al. demonstrated that substituting the VP3 C-terminal region of a serotype I vvIBDV with that of serotype II significantly reduced morbidity and mortality in vaccinated chickens [73]. Using an attenuated Gt strain as a backbone, Gao et al. generated a chimeric *r*GtHLJVP2, by replacing its VP2 with that of vvIBDV HLJ0504, which induced antibody titers comparable to the live attenuated vaccine B87, caused no clinical or pathological lesions in SPF chickens [74]. Gao et al. also found that the N-terminal domain of VP1 from vvIBDVs had a negative effect on viral replication in vitro while exhibiting a positive effect on viral replication in vivo [75]. However, Nouen et al. found that the association of the central polymerase domain (Dc) and non-coding regions (NCRs) at the 5′ and 3′ ends of the 88180 strain increased the titer of virus at 4 dpi, but it did not result in an increased pathogenicity in chickens [76]. Wang et al. demonstrated that the full region of the N-terminal of polymerase plays an important role in viral replication and pathogenicity, but the substitutions of its partial region or a single residual do not completely lead to the virus attenuation to Three-Yellow chickens, although that significantly reduces its pathogenicity [77]. More recently, Yang et al. reported that insertion of a FLAG tag at the VP1 C-terminus markedly reduced polymerase activity and virulence, highlighting a novel strategy for rapidly development of antigen-matched live vaccines against currently circulating nvIBDV strains [78].

## 8. Nucleic Acid Vaccines

### 8.1. DNA Vaccines

Jiang et al. developed DNA vaccines based on IBDV VP2 and VP3, with protection rates of 90% and 10% in chickens, respectively [79]. Fodor et al. directly muscularly immunized SPF chickens with a DNA vaccine constructed based on the polyprotein VP2/4/3 [80]. Although the VP2/4/3 polyprotein induced immune responses, only 55% of chickens were antibody-positive, and the overall protection rate was just 36%. Li et al. developed an oral DNA vaccine based on the VP2/4/3 polyprotein gene delivered by attenuated *Salmonella Typhimurium*, which elicited specific humoral responses in SPF chickens [81]. However, even after booster immunization with an inactivated vaccine, the protection rate reached only 73.3%. Mahmood et al. developed an oral DNA vaccine (EC/pRc-VP2) containing the vvIBDV VP2 gene delivered by *E. coli* DH5α, which provided 95.4% protection in chickens against the vvIBDV challenge, significantly higher than the protection achieved by the commercial attenuated D78 vaccine [82]. A prime-boost regimen, using a DNA vaccine for priming (in ovo or at day one) and an inactivated or vectored vaccine for boosting, confers protection in chickens, whereas in ovo DNA vaccination alone proves not sufficient to induce protective immunity. Park et al. immunized chicken embryos with a DNA vaccine containing the polyprotein VP2/4/3 gene, either alone or in combination with plasmids encoding IL-2 and chIFN-γ, followed by a booster with inactivated vaccine at one day post-hatch [83]. They found that the prime-boost strategy using the DNA vaccine and inactivated vaccine completely protected chickens against vvIBDV challenge, whereas the addition of chIL-2 or chIFN-γ did not enhance the protection rate. Negash et al. evaluated the efficacy of a cationic polymer PLGA-MP for delivering an IBDV-VP2 DNA vaccine in chickens and demonstrated that PLGA-MP significantly enhanced the immunogenicity of the VP2 DNA vaccine [84]. Moreover, other studies have reported that IL-2 [85] and IL-6 [86] can markedly improve the efficacy of DNA vaccines. To date, DNA vaccines against IBDV remain at the experimental research stage and have not yet been licensed for commercial use.

### 8.2. RNA Vaccines

RNA vaccines have become highly attractive in recent years. Early studies demonstrated that naked mRNA used as an immunogen can rapidly induce both humoral and cellular immune responses. However, initial development was constrained by challenges such as high production costs, instability during long-term storage and in vivo delivery, and complex manufacturing processes. Recent advances have shown that stable mRNA vaccines against infectious diseases can be produced under GMP conditions with high immunogenicity, which has driven the rapid development of mRNA vaccine technology. Currently, several mRNA-based SARS-CoV-2 vaccines have been approved for clinical use, including Pfizer’s BNT162b2, Moderna’s mRNA-1273, and Janssen’s Ad26.COV2.S. Both the Pfizer and Moderna vaccines require a two-dose regimen and storage at −20 °C to −70 °C, whereas the Janssen vaccine is approved as a single-dose formulation and can be stored for several months at 4 °C. To date, all SARS-CoV-2 vaccines target the original or optimized spike protein of the virus using various platforms, with over 50 RNA vaccine candidates in different stages of clinical trials. Current mRNA vaccine development efforts largely focus on preventing diseases, including human immunodeficiency virus (NCT05001373), influenza [87], rabies virus [88], Zika virus (NCT04064905), respiratory syncytial virus (NCT04528719), and cytomegalovirus (NCT04232280). Only a few mRNA vaccines have been developed for veterinary use, such as those against foot-and-mouth disease virus [89]. In 2022, Qu et al. reported a circular mRNA vaccine, circRNARBD-Delta, which demonstrates greater stability and resistance to exonuclease degradation compared to linear mRNA vaccines, and showed advantages in production, delivery, and therapeutic efficacy [90]. In 2024, Chen et al. further improved mRNA vaccine performance by engineering a novel structure with multiple poly (A) tails, significantly enhancing translation efficiency and stability [91]. Although challenges remain for the application of mRNA vaccines in veterinary medicine, their future potential is considerable. Promising directions include T cell-targeted mRNA vaccines against African swine fever virus (ASFV), VP2-based multivalent chimeric mRNA vaccines for IBDV and porcine reproductive and respiratory syndrome virus (PRRSV), RBD-based mRNA vaccines against infectious bronchitis virus (IBV), and E2-based vaccines against swine fever virus.

To date, no licensed or experimentally validated mRNA vaccine specifically targeting IBDV has been reported. Nevertheless, we included a detailed overview of mRNA and saRNA vaccine technologies because these platforms have demonstrated rapid, safe, and effective antigen expression for multiple avian and mammalian viral pathogens, and they represent a promising next-generation strategy for IBDV control.

### 8.3. Self-Amplifying Vaccines

Self-amplifying vaccines, originating from the genomes of alphavirus, include Semliki Forest virus (SFV), Sindbis virus (SINV), and Venezuelan equine encephalitis virus (VEEV) [92,93]. Self-amplifying vaccine contains two open reading frames (ORFs). The first ORF encodes four non-structural proteins (nsP1-4), which were responsible for the replication and amplification of the viral genome. The second ORF encodes an exogenous target antigen, which is expressed through sub-genomic RNA generated by replicase-driven transcription initiated at the viral 26S sub-genomic promoter, enabling continuous and large-scale expression of the target antigen. Unlike conventional non-amplifying nucleic acid vaccines that only encode the target antigen gene, self-amplifying vaccines can achieve prolonged and enhanced antigen expression even at very low RNA doses [94], thereby inducing stronger humoral and cellular immune responses [95]. Recently, the first fully licensed self-amplifying nucleic acid vaccine in the world, ARCT-154, developed by Arcturus Therapeutics (USA) and CSL (Japan), and was approved by Japanese health authorities [96]. Additionally, in 2022, India approved a self-amplifying vaccine named Gemcovac-19, produced by Gennova Biopharmaceuticals (Pune, India), for emergency use only. Jerome et al. developed a bird-adapted VEEV-based self-amplifying mRNA versatile vaccine platform successfully expressed AIV HA and IBDV pVP2 proteins, generating high levels neutralizing antibodies in poultry and highlighting the significant potential against emerging pathogens [97]. Currently, self-amplifying nucleic acid vaccines have been applied to various emerging or re-emerging infectious diseases in both humans and animals, such as SARS-CoV-2 [98], HIV-1 [99], influenza virus [94], rabies virus (NCT04062669), Zika virus [100], respiratory syncytial virus [101], botulism [102], foot-and-mouth disease virus [103], classical swine fever virus [104], and goatpox virus [105].

## 9. Field Performance and Application of IBDV Vaccines

While technological innovation in vaccine platforms provides the tools for control, under field conditions, the performance of infectious BF disease virus (IBDV) vaccines is shaped not only by antigenic compatibility and formulation but also by poultry management, biosecurity standards, and interference from MDA. Commercial broilers are particularly susceptible to early infection when MDA levels decline, co-infections with other pathogens or an environment where high viruses circulate. Previous studies indicate that live attenuated vaccines induce immunity rapidly but are strongly affected by MDA, while inactivated and Icx vaccines provide slower yet more consistent protection under high-MDA conditions [14]. Recombinant HVT-VP2 vaccines confer long-lasting immunity with minimal MDA interference, making them suitable for large-scale hatchery use. Ultimately, the selection of vaccine type should be guided by Prevalent Genotypes and Antigenic Types, Antigenic Drift and Emerging Variants, and MDA interference, which remains the cornerstone strategy for preventing IBD-associated immunosuppression in commercial poultry.

## 10. Breeder Hen Vaccination and MDA Dynamics

Vaccination of breeder hens plays a pivotal role in providing passive immunity to progeny. The primary objective is to elicit high titers of neutralizing antibodies in hens that are efficiently transferred to chicks via the egg yolk, offering early protection during the critical post-hatch period [19]. These MDA protect chicks for 2–3 weeks, bridging the gap before their immune systems mature. The duration and level of MDA are influenced by vaccine formulation, adjuvant choice, and booster timing. Oil-emulsified inactivated vaccines are most commonly used in breeders due to their prolonged antibody persistence. The breeder vaccination protocols typically employ a prime-boost regimen, utilizing live vaccines followed by inactivated, oil-adjuvanted vaccines prior to the onset of lay to ensure persistent and high-level antibody production [14,30]. While robust MDA is indispensable for early protection, excessively high-MDA titers can interfere with early progeny vaccination [19,20]. Therefore, precise scheduling of breeder vaccination is required to synchronize MDA decay with the onset of active immunity in chicks, optimizing flock-level protection.

## 11. The Critical Age Window for BF Protection and Time to Antibody Induction by Vaccine Type

Protection of the bursa of Fabricius is essential between 14 and 35 days of age, when B-cell development is most active. Infection during this period can result in irreversible immunosuppression, poor vaccine responsiveness, and increased susceptibility to secondary infections. Effective vaccination aims to induce protective antibody levels before 14 days of age, especially in regions where very virulent (vvIBDV) or novel variant IBDV (nvIBDV) strains are prevalent. Under conditions of high-challenge pressure, early immunization through Icx or recombinant vector vaccines provides extended coverage throughout this critical developmental window (Table 2).

## 12. Early Challenge and Reused Litter Conditions

In broiler production systems that reuse litter, residual IBDV can persist between flocks, leading to early infection as soon as 5–7 days post-hatch. Such early exposure frequently occurs before active immunity develops. Icx and recombinant vectored vaccines are particularly suitable for these environments, as they are less affected by MDA and provide early protection through mechanisms such as delayed virus release and sustained antigen presentation [34,35,36,37,44]. Conversely, conventional live attenuated vaccines may fail due to rapid neutralization by MDA. Robust breeder vaccination remains an indispensable adjunct to protect chicks during this vulnerable period.

## 13. Vaccination Necessity Under Low-Challenge Environments

In poultry operations employing single-flock litter replacement and maintaining high biosecurity, the environmental load of IBDV is significantly reduced. In such low-challenge conditions, active broiler vaccination may be unnecessary if breeder hens have been effectively immunized. Decision-making should consider factors such as historical farm challenge rates, presence of vvIBDV or nvIBDV strains, flock density, and cost–benefit analysis. In low-risk systems, reliance on passive immunity from breeder vaccination combined with strict hygiene can be both effective and economically advantageous. Nonetheless, sentinel monitoring and periodic serological surveillance are recommended to ensure continued protection and early detection of viral re-emergence.

## 14. Conclusions and Future Directions

The continuous evolution of IBDV, particularly the emerge of very virulent and antigenic variant strains, poses a significant challenge to the conventional vaccination strategies. While traditional live-attenuated and inactivated vaccines have played a crucial role in controlling IBD, their limitations, such as MDA interference, potential reversion to virulence, and insufficient cross-protection, highlight the need for more advanced vaccine platforms to develop new types of vaccines that can keep pace with the evolution of viruses.

Effective IBDV vaccination strategies must be tailored to the epidemiological context and the management and biosecurity programs in which they are applied. In commercial broilers, protection is determined not only by the intrinsic efficacy of the vaccine strain but also by MDA interference, timing of administration, and challenge pressure. MDA plays a critical role in providing early immunity to chicks but can also interfere with the induction of active immunity, especially when MDA levels are too high. In systems with high MDA, immune-complex vaccines (rapid immune activation), recombinant vectored vaccines and subunit vaccines (offer safer and longer-lasting immunity) show promise by offering protection despite MDA interference. Nevertheless, the choice of vaccine should reflect local virus circulation patterns, litter management, and biosecurity standards.

Breeder hen vaccination remains central to flock protection, as MDA transfer shields chicks during their first 2–3 weeks of life—when they are most susceptible to infection. The balance between robust MDA levels and the timing of broiler vaccination is critical, as excessive MDA may delay active immune induction. Under high-challenge conditions or when litter is reused, immune-complex and recombinant vectored vaccines are preferred because they confer earlier and more consistent protection despite MDA interference. Conversely, in low-risk environments with single-flock litter replacement and stringent biosecurity, reliance on breeder immunization and environmental control may be sufficient, avoiding unnecessary vaccination costs. The critical window for protecting the bursa of Fabricius, generally between 14 and 35 days of age, must be considered when designing vaccination programs. Damage to the bursa during this stage results in irreversible immunosuppression and decreased responsiveness to other poultry vaccines. The integration of vaccine type, administration timing, and local risk assessment is therefore essential for sustainable disease control.

Moreover, gene editing technologies, such as reverse genetics systems and CRISPR/Cas9, offer exciting potential for developing precision vaccines against IBDV. By enabling precise modifications to viral antigens, gene editing technologies can help create vaccines that are tailored to emerging viral strains and variants. This technology allows for rapid adaptation of vaccines to new IBDV strains, potentially overcoming challenges such as antigenic drift and improving cross-protection. The application of gene editing technologies in vaccine development is still in its early stages, but it holds promise as a tool for generating highly specific vaccines that address the evolving challenges posed by IBDV.

At present, mRNA vaccines have not yet been developed or validated for IBDV; however, their rapid design cycle, strong immunogenicity, and flexible antigen-encoding capacity make them an attractive platform for future research. Advances achieved in mRNA vaccines for other avian and mammalian pathogens could be readily leveraged for IBDV, particularly for generating VP2 antigen constructs capable of overcoming antigenic drift and maternal antibody interference. Future work should evaluate delivery systems suitable for poultry, thermostability improvements for field conditions, and in vivo expression efficiency in the bursa-associated immune environment.

Looking ahead, future IBDV vaccine development should aim to combine strong early protection, broad antigenic coverage, and compatibility with hatchery-based delivery systems such as in ovo or spray vaccination. The continuous antigenic drift of IBDV necessitates ongoing surveillance and timely updates to vaccine strains. Recombinant vectored vaccines, reverse genetics, mRNA platforms, and multivalent recombinant constructs represent promising directions for next-generation vaccines. Ultimately, the success of IBD control depends not only on vaccine innovation but also on the rational application of vaccination within comprehensive management frameworks that consider MDA dynamics, environmental challenge, and production economics.

## Figures and Tables

**Table 1:** Chronology of IBDV vaccine development.

Vaccine types	Years (R&D or License)	Key Milestone	Representative Product and Manufacturer
Live Attenuated IBDV Vaccine	1980s	The first commercial live attenuated vaccines were introduced for poultry, offering a more practical solution to IBDV control.	Bursine^®^ (Zoetis, Parsippany, NJ, USA), CEVAC^®^ IBD L (Strain W2512), CEVAC^®^ GUMBO L strain LIBDV (Ceva, Libourne, France), NOBILIS^®^ GUMBORO D78 (MSD, Boxmeer, The Netherlands), Bur-Cell^®^ 2 + 0 (Standard and Variant), Bursa-Blen^TM^ M (Boehringer Ingelheim, Ingelheim am Rhein, Germany), B87 CVCAV140 (Meilan Bio, Nanjing, China), Strain B87 + Strain CA + Strain CF (Harbin Bio-Vaccine, Harbin, China)
Inactivated IBDV Vaccine	1980s	Early inactivated vaccine for IBDV, widely used in large-scale poultry operations for IBDV control	CEVAC^®^ ND IB IBD K (Ceva), Bursa Guard^TM^ N-B-R, and Bursa Guard^TM^ Reo (Boehringer Ingelheim), Strain La Sota + Strain M41 + Strain BJQ902 + Strain WD (Keqian Bio, Wuhan, China)
DNA vaccine	1990s	VP2 was confirmed as the key immunogenic protein responsible for neutralization; expression of VP2 alone could elicit protective immunity.	pVP2 (Academic research)
Immune Complex Vaccine (Icx)	1990s	The first commercial ICX vaccine; uses intermediate IBDV strain complexed with specific antibodies	BDA-Blen^TM^ (Boehringer Ingelheim), Bursa-Vac^®^ (Merck, Madison, NJ, USA), Transmune^®^ IBD (Ceva), Poulvac^®^ Bursaplex^®^ (Zoetis)
Recombinant Subunit Vaccine (VP2/VLP)	2010s	Combining VP2 subunit vaccines with inactivated vaccines allows for more efficient vaccination protocols in poultry, reducing the need for multiple separate vaccinations.	ND-IB-AI-IBD (Strain La Sota + Strain M41 + Strain SZ + Protein rVP2), NDV-IFV-IBDV-FAdV (Strain N7a + Strain HF + Protein rVP2 + Protein Fiber-2) approved for veterinary use in Henan,China
Recombinant Live Virus Vector Vaccine	2010s	First licensed IBDV recombinant vector vaccine, dual protection (MD + IBD).	Vaxxitek^®^ HVT + IBD, VAXXITEK^®^ HVT + IBD + ILT, VAXXITEK^®^ HVT + IBD + ND (Boehringer Ingelheim), Innovax^®^ ND-IBD, Innovax^®^ ILT-IBD, and Innovax^®^ ND-IBD-ILT (MSD), VECTORMUNE^®^ HVT-IBD (Ceva)
Reverse-genetics-derived recombinant live vaccines	2010s	Advancement of reverse-genetics-derived IBDV vaccines with rationally designed attenuated entering commercial evaluation and regulatory review.	attenuated rgIBDV (Academic research)
mRNA/saRNA IBDV vaccines	2020s	Introduction of mRNA and self-amplifying RNA (saRNA) vaccine platforms targeting IBDV with validated LNP delivery, and enhanced immunogenicity at lower doses demonstrated in SPF chickens.	saRNA-VP2, LNP-VP2 mRNA (Academic research)

**Table 2:** Comparative characteristics across major IBDV vaccine types.

Vaccine Type	Onset of Detectable Immune Response	Typical Time to Protective Immunity	Duration of Protection	Advantages and Limitations
Live attenuated	3–5	7–10	3–4 weeks	Rapid onset, sensitive to MDA interference, “occupancy” of the bursa, humoral & cellular immunity [21,22]; Antigenic prone to drift; Risk of reversion to virulence; Production cost relatively low.
Inactivated	7–10 (after booster)	14–21 (post-booster)	>8 weeks	Requires adjuvant and booster dosing, primarily strong humoral immunity (IgG) [14,30,31,32]; Antigenic stability high; easier regulation; Production cost moderate.
Subunit vaccine	10–14	21–28	Variable, (boost-dependent)	Requires strong adjuvant, usually as booster, high safety, suitable for breeders and progeny [54,61,62]; Antigenic specificity; Easier to approve; Production cost high
Immune complex	14–17	14–28	Moderate	Delayed virus release, targeted delivery to immune cells, Effective in high-MDA [33,34,35,36,37]; Field Effectiveness very high, early protection; risk of drift; Production cost high due to complex preparation.
rHVT-VP2 vectored	7–10	14–21	Long-lasting	Continuous antigen expression, strong humoral & cellular immunity, minimal interference from MDA [38,41,42,43]; effective in MDA conditions; Vector immune pressure; High regulation due to genetic modification; Production cost high
DNA/mRNA	7–10 (post-booster)	14–21 (post-booster)	Variable	Endogenous antigen expression, humoral & cellular immunity, requires adjuvant and delivery [80,81,82,83,84,85,86]; Effectiveness unproven in field; Antigenic stability high, adaptable to variants; Production cost high

## Data Availability

No new data were created or analyzed in this study. Data sharing is not applicable to this article.
